# Author Correction: ZIP1^+^ fibroblasts protect lung cancer against chemotherapy via connexin-43 mediated intercellular Zn^2+^ transfer

**DOI:** 10.1038/s41467-023-36663-1

**Published:** 2023-02-16

**Authors:** Chen Ni, Xiaohan Lou, Xiaohan Yao, Linlin Wang, Jiajia Wan, Xixi Duan, Jialu Liang, Kaili Zhang, Yuanyuan Yang, Li Zhang, Chanjun Sun, Zhenzhen Li, Ming Wang, Linyu Zhu, Dekang Lv, Zhihai Qin

**Affiliations:** 1 Medical Research Center, the First Affiliated Hospital of Zhengzhou University, 450052 Zhengzhou, China; 2 Institute of Cancer Stem Cell, Dalian Medical University, 116044 Dalian, China; 3 Institute of Biophysics, Chinese Academy of Sciences, 100101 Beijing, China

**Keywords:** Cancer microenvironment, Non-small-cell lung cancer, Cancer therapeutic resistance, Chemotherapy

Correction to: *Nature Communications* 10.1038/s41467-022-33521-4, published online 07 October 2022

The original Article contained an error in the Results section “ZIP1+ fibroblasts are enriched in mouse lung cancer after chemotherapy”. The third sentence of this paragraph incorrectly read “Lewis lung carcinoma (LLC) cells with ubiquitous enhanced green fluorescent protein (EGFP) expression were subcutaneously transplanted into C57BL/6 mice.” in the original text.

The current sentence is: “Lewis lung carcinoma (LLC) cells were subcutaneously transplanted into C57BL/6 mice with ubiquitous enhanced green fluorescent protein (EGFP) expression”.

The sentence has been corrected in the PDF and HTML versions of the Article.

