# Supplementary material for: ZIP1+ fibroblasts protect lung cancer against chemotherapy via connexin-43 mediated intercellular Zn2+ transfer
Source: Nat Commun. 2022 Oct 7;13:5919. doi: 10.1038/s41467-022-33521-4 (PMC9547061; doi:10.1038/s41467-022-33521-4)
Supplement: Supplementary file 1 — Supplementary Information [file 41467_2022_33521_MOESM1_ESM.pdf]

Supplementary Information

ZIP1^{+} fibroblasts protect lung cancer against chemotherapy via connexin-43 mediated intercellular Zn^{2+} transfer

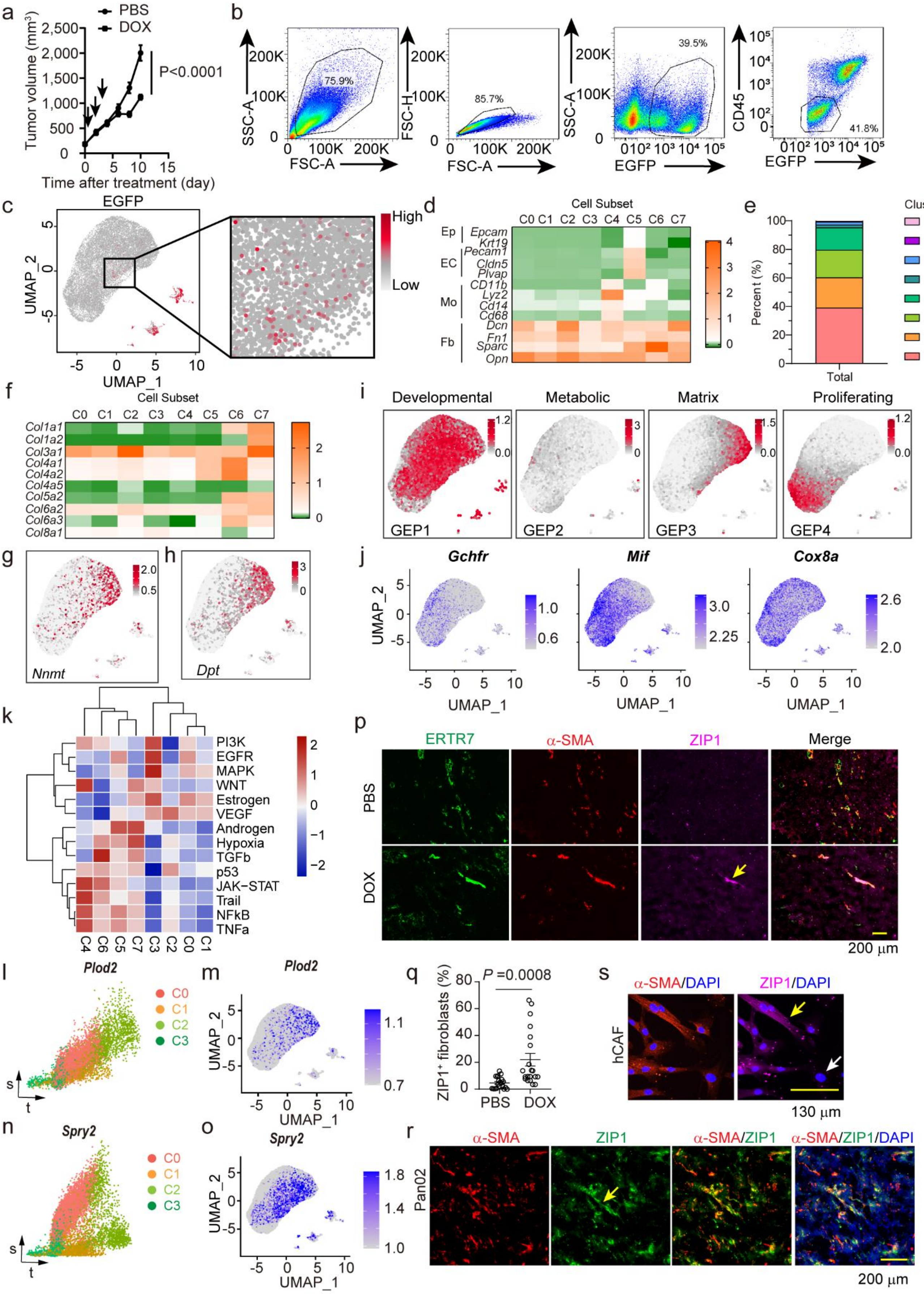
a
PBS
DOX
Tumor volume (mm³)
P<0.0001
Time after treatment (day)
b
SSC-A
FSC-A
75.9%
FSC-H
85.7%
39.5%
EGFP
CD45
41.8%
c
EGFP
UMAP_2
UMAP_1
High
Low
d
Cell Subset
C0 C1 C2 C3 C4 C5 C6 C7
Ep
Epcam
Krt19
EC
Pecam1
Cldn5
Plvap
Mo
CD11b
Lyz2
Cd14
Cd68
Fb
Dcn
Fn1
Sparc
Opn
e
Cluster:
C7
C6
C5
C4
C3
C2
C1
C0
Percent (%)
Total
f
Cell Subset
Col1a1
Col1a2
Col3a1
Col4a1
Col4a2
Col4a5
Col5a2
Col6a2
Col6a3
Col8a1
g
Nnmt
h
Dpt
i
Developmental
Metabolic
Matrix
Proliferating
GEP1
GEP2
GEP3
GEP4
j
Gchfr
Mif
Cox8a
k
PI3K
EGFR
MAPK
WNT
Estrogen
VEGF
Androgen
Hypoxia
TGFb
p53
JAK-STAT
Trail
NFkB
TNFa
C4 C6 C5 C7 C3 C2 C0 C1
l
Plod2
m
Plod2
n
Spry2
o
Spry2
p
ERTR7
α-SMA
ZIP1
Merge
PBS
DOX
200 μm
q
ZIP1⁺ fibroblasts (%)
P =0.0008
PBS
DOX
r
α-SMA
ZIP1
α-SMA/ZIP1
α-SMA/ZIP1/DAPI
Pan02
200 μm
s
α-SMA/DAPI
ZIP1/DAPI
hCAF
130 μm

Supplementary Figure 1. scRNAseq shows that *Zip1*⁺ fibroblasts are enriched in LLC tumors after DOX treatment. (**a**) Growth curves of LLC tumor implanted into C57BL/6 mice without or with DOX (5 mg/kg) treatment (black arrow, at day 0, 2, 4). PBS, n = 5; DOX, n =6. Two-way ANOVA for tumor growth curve comparison. (**b**) Sorting strategy of $EGFP^+CD45^−$ stromal cells for scRNAseq. (**c**) UMAP plot showing the expression of EGFP in recovered cells. (**d**) Heatmap showing cell-specific makers of each subset. Ep, epithelial cell; EC, endothelial cell; Mo, monocyte; Fb, fibroblast. (**e**) Faceted bar graph showing the cell percentages of cell subsets. (**f**) Heatmap showing the expression of collagens in cell subsets. (**g**) UMAP plot showing the expression of *Nnmt* in cell subsets. (**h**) UMAP plot showing the expression of *Dpt* in cell subsets. (**i**) UMAP plot showing the activity of gene expression programs 1-4 (GEP1-4) in cell subsets. (**j**) UMAP plot showing the expression of *Gchfr*, *Mif* and *Cox8a* in cell subsets. (**k**) Pathway activity as estimated by the PROGENy algorithm. (**l**) Changes of spliced (s) mRNAs of *Plod2* along with latent time (t) estimated with scVelo. (**m**) UMAP plot showing the expression of *Plod2* in cell subsets. (**n**) Changes of spliced (s) mRNAs of *Spry2* along with latent time (t) estimated with scVelo. (**o**) UMAP plot showing the expression of *Spry2* in cell subsets. (**p**) LLC tumor sections from mice treated with PBS or DOX were stained for ZIP1 and the fibroblast marker ERTR7/α-SMA. Arrow, $ZIP1^+$ fibroblasts. Representative staining results from three tumours for each group are shown. (**q**) The percentage of $ZIP1^+$ fibroblasts was determined by comparing ZIP1/ERTR7 co-expression to the ERTR7 signal in LLC tumors. Mann–Whitney test for two-group comparison. Twenty images derived from three tumours for each group were analysed. (**r**) Pan02 tumor sections were stained for ZIP1 and the fibroblast marker α-SMA. Arrow, $ZIP1^+$ fibroblasts. Representative staining results from two independent experiments are shown. (**s**) Immunostaining of ZIP1 and α-SMA in human lung adenocarcinoma-associated fibroblasts (hCAF). Yellow arrow, $ZIP1^+$ fibroblasts; white arrow, $ZIP1^-$ fibroblasts. Representative staining results from two independent experiments are shown. Source data are provided as a Source Data file (**a, e, q**).

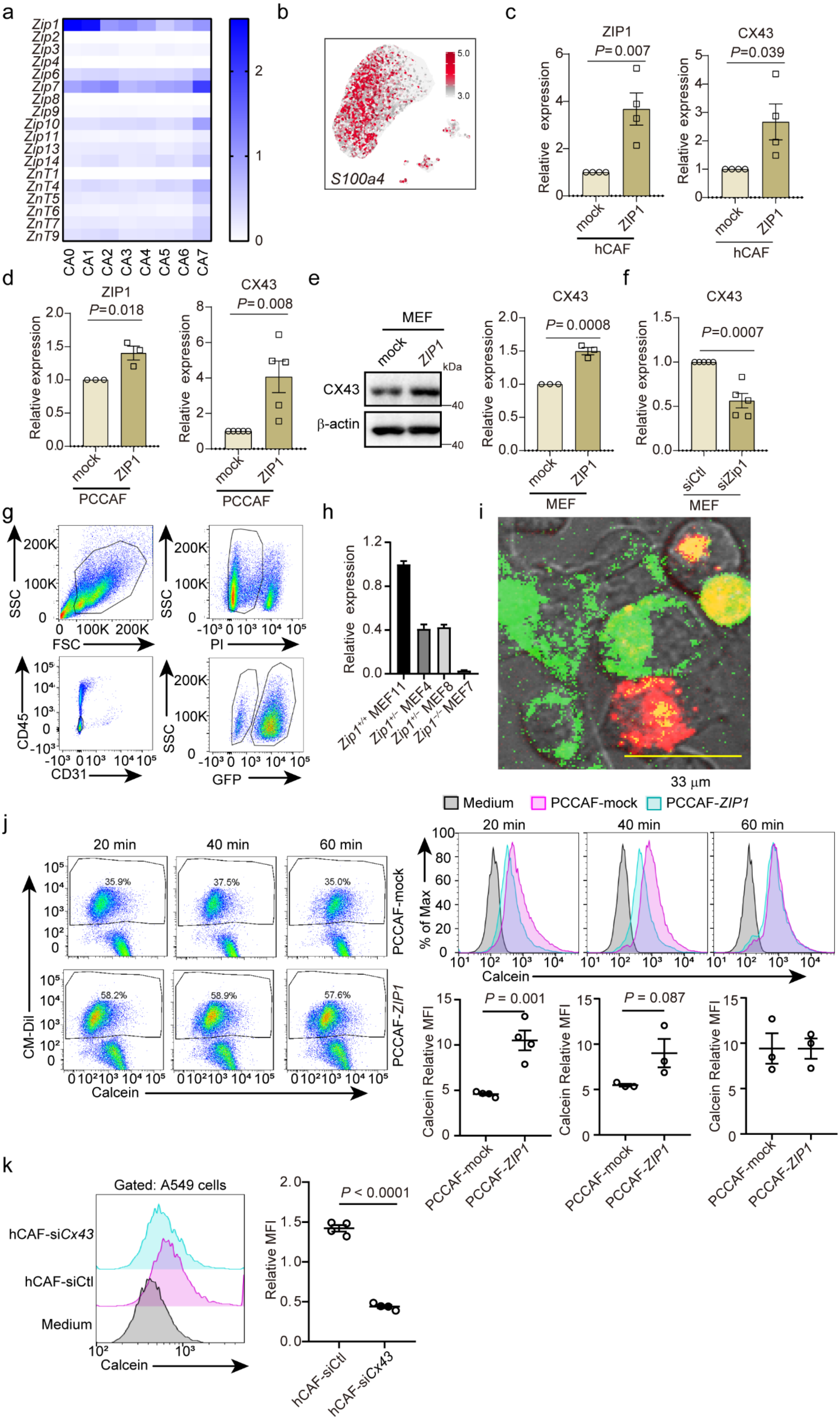
a
Zip1
Zip2
Zip3
Zip4
Zip6
Zip7
Zip8
Zip9
Zip10
Zip11
Zip13
Zip14
ZnT1
ZnT4
ZnT5
ZnT6
ZnT7
ZnT9
CA0 CA1 CA2 CA3 CA4 CA5 CA6 CA7
2
1
0
b
S100a4
5.0
3.0
c
ZIP1
P=0.007
CX43
P=0.039
Relative expression
mock ZIP1
hCAF
d
ZIP1
P=0.018
CX43
P=0.008
Relative expression
mock ZIP1
PCCAF
e
MEF
mock ZIP1
kDa
CX43
β-actin
40
40
CX43
P= 0.0008
Relative expression
mock ZIP1
MEF
f
CX43
P=0.0007
Relative expression
siCtl siZip1
MEF
g
SSC
FSC
PI
CD45
CD31
GFP
h
Relative expression
Zip1+/+ MEF11
Zip1+/- MEF4
Zip1+/- MEF8
Zip1-/- MEF7
i
33 μm
j
20 min
40 min
60 min
35.9%
37.5%
35.0%
58.2%
58.9%
57.6%
PCCAF-mock
PCCAF-ZIP1
CM-Dil
Calcein
Medium
PCCAF-mock
PCCAF-ZIP1
% of Max
Calcein
P = 0.001
P = 0.087
Calcein Relative MFI
PCCAF-mock PCCAF-ZIP1
k
Gated: A549 cells
hCAF-siCx43
hCAF-siCtl
Medium
Calcein
P < 0.0001
Relative MFI
hCAF-siCtl hCAF-siCx43

Supplementary Figure 2. ZIP1$^+$ fibroblasts interconnect lung cancer cells with gap junctions. (**a**) Heatmap showing the expression of zinc transporters by fibroblast subtypes. Single-cell RNA sequencing data derived from mixed three tumour samples for both PBS and DOX group. (**b**) UMAP plot showing the expression of *S100a4* in cell subsets. Single-cell RNA sequencing data derived from mixed three tumour samples for both PBS and DOX group. **(c)** Quantification of ZIP1 and CX43 expression detected by Western-blotting in **Fig.2d** from four independent experiments. **(d)** Quantification of ZIP1 and CX43 expression detected by Western-blotting in **Fig.2e** from three (ZIP1) or five (CX43) independent experiments. (**e**) Western-blotting determination of expression of CX43 in mouse embryonic fibroblasts (MEFs) transfected with control (mock) or ZIP1-overexpression vector (*ZIP1*). Quantification from three independent experiments is shown. **(f)** Quantification of ZIP1 and CX43 expression detected by Western-blotting in **Fig.2f** from five independent experiments. (**g**) Gating strategy for CD45$^-$CD31$^-$GFP$^-$ CAFs from LLC-GFP-luc tumor transplanted in *Zip1*$^{+/+}$, *Zip1*$^{+/-}$ and *Zip1*$^{-/-}$ mice on day 22. n = 3 for *Zip1*$^{+/+}$, n = 5 for *Zip1*$^{+/-}$ and *Zip1*$^{-/-}$. (**h**) Expression of *Zip1* in *Zip1*$^{+/+}$, *Zip1*$^{+/-}$ and *Zip1*$^{-/-}$ mouse embryonic fibroblasts (MEFs) was determined by qPCR. n = 3 for each group. (**i**) PCCAF-CX43-GFP and A549-CX43-RFP co-culture showed physical contacts between CX43-GFP and CX43-RFP. A representative result from three independent experiments is shown. (**j**) Calcein transfer between PCCAFs and A549 tumor cells. A549 cells were labeled with CM-Dil. PCCAF-mock or PCCAF-*ZIP1* were loaded with calcein-AM. Fibroblasts were co-cultured with A549 for different times in DMEM+10%FBS. Medium: A549 alone without fibroblasts used as control. n = 4 for PCCAF-mock, PCCAF-*ZIP1* 20 min, n = 3 for PCCAF-mock, PCCAF-*ZIP1* 40 min, 60 min. (**k**) Calcein transfer between hCAF-si*Cx43* and A549 tumor cells. Medium: A549 alone. Gating strategy similar to (**j**) and A549 cells were gated. n = 4 for each group. Unpaired *t*-test for two group comparison. Source data are provided as a Source Data file (**a, c-f, h, j-k**).

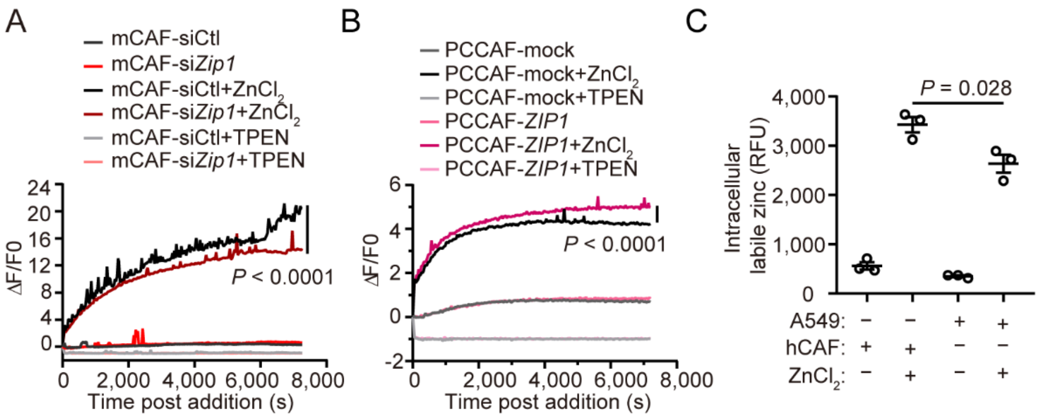

Supplementary Figure 3. ZIP1 expression promotes Zn^{2+} absorption in fibroblasts. (**a**) Zn^{2+} uptake by mouse cancer-associated fibroblasts (mCAFs) transfected with control (siCtl) or *Zip1*-silencing (si*Zip1*) siRNA, ± $ZnCl_2$ (30 µM) or TPEN chelator (2 µM) in HBSS. n = 3 for each group. (**b**) Zn^{2+} uptake by prostate-cancer CAFs (PCCAFs) transfected with control (mock) or ZIP1-overexpression vector (*ZIP1*), ± $ZnCl_2$ or TPEN chelator. n = 3 for each group. (**c**) Labile Zn^{2+} levels in hCAFs and A549 tumor cells with or without addition of $ZnCl_2$. A549 or hCAF cells were cultured overnight and loaded with FluoZin3-AM. $ZnCl_2$ (30 µM) was added in DMEM for 30 min. After changing DMEM to HBSS, the FluoZin3 fluorescence was read. RFU, relative fluorescence units. n = 3 for each group. Unpaired *t*-test for two group comparison. Two-way ANOVA for tumor growth curve comparison. Source data are provided as a Source Data file (**a-c**).

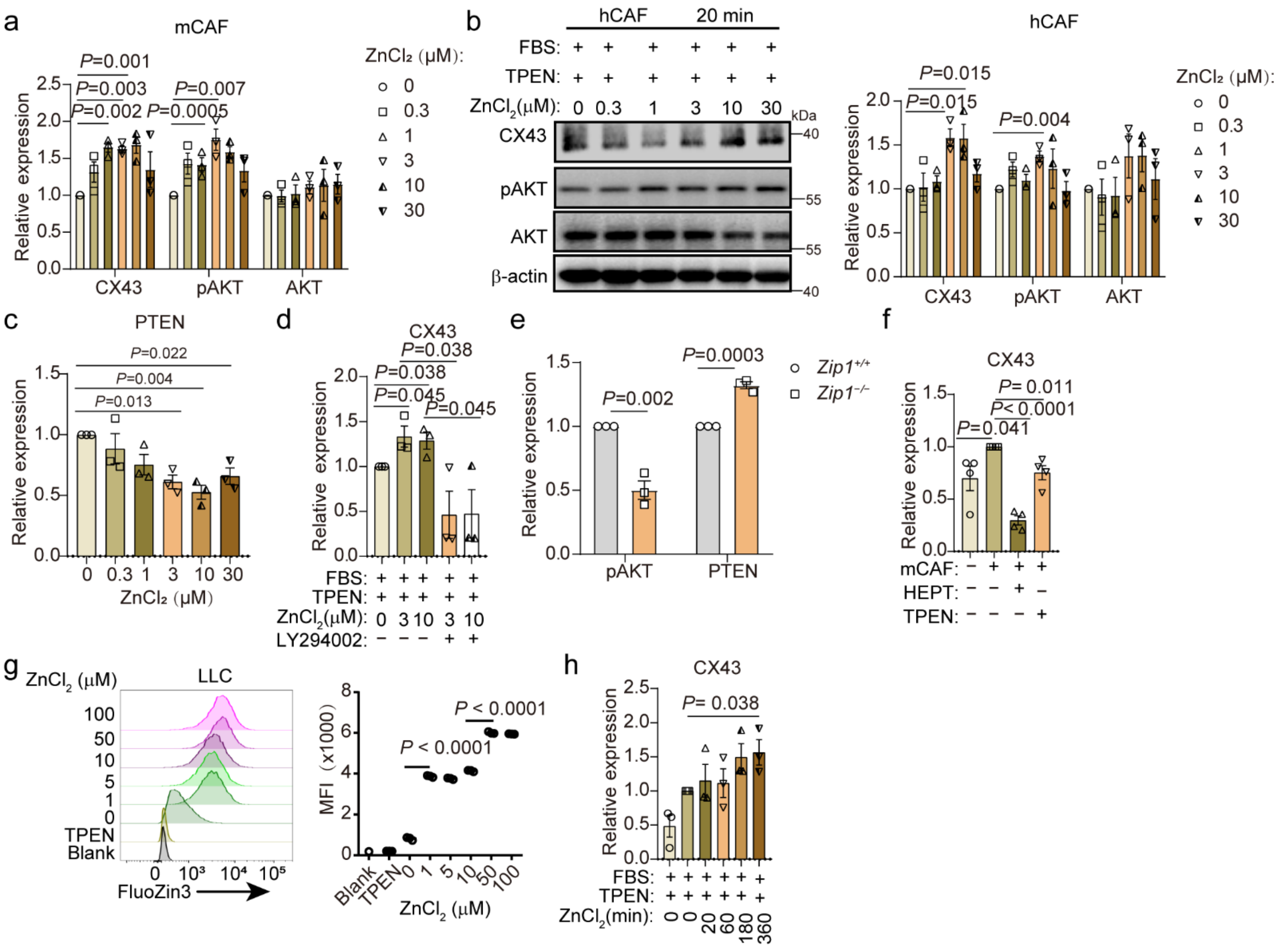

Supplementary Figure 4. Labile Zn^{2+} upregulates CX43. (a) Quantification of CX43, pAKT and AKT expression detected by Western-blotting in **Fig.4a** from three independent experiments. (**b**) Labile Zn^{2+} in LLC cells after addition of different concentrations of $ZnCl_2$ in DMEM+10%FBS+5 μM TPEN for 20 min. Quantifications from three independent experiments are shown. LLC cells were pre-loaded with FluoZin3-AM. Blank, no FluoZin3-AM. TPEN, LLC with FluoZin3 were treated with 5 μM TPEN in FACS buffer. **(c)** Quantification of PTEN expression detected by Western-blotting in **Fig.4b** from three independent experiments. **(d)** Quantification of CX43 expression detected by Western-blotting in **Fig.4c** from three independent experiments. **(e)** Quantification of pAKT and PTEN expression detected by Western-blotting in **Fig.4d** from three independent experiments. **(f)** Quantification of CX43 expression detected by Western-blotting in **Fig.4e** from four independent experiments. (**g**) Labile Zn^{2+} in LLC cells after addition of different concentrations of $ZnCl_2$ in DMEM+10%FBS+5 μM TPEN for 20 min. LLC cells were pre-loaded with FluoZin3-AM. Blank, no FluoZin3-AM. TPEN, LLC with FluoZin3 were treated with 5 μM TPEN in FACS buffer. n = 3 for each group. **(h)** Quantification of CX43, pAKT and AKT expression detected by Western-blotting in **Fig.4f** from three independent experiments. Unpaired *t*-test for two group comparison. One-way ANOVA with Tukey's *post hoc* analysis for multigroup comparisons. Source data are provided as a Source Data file (**a-h**).

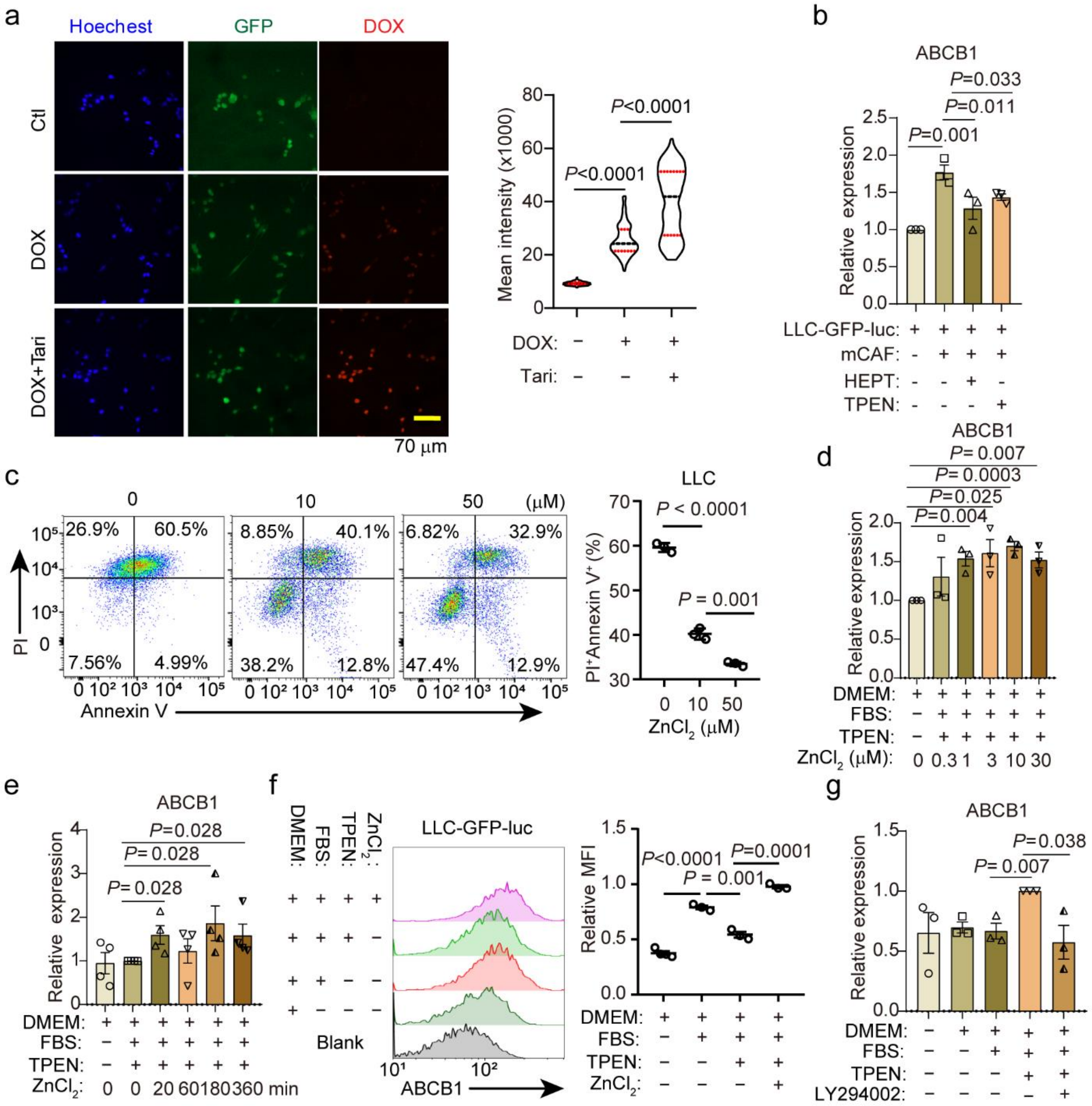

Supplementary Figure 5. Labile Zn^{2+} protects tumor cells from drug toxicity. (**a**) DOX accumulation in LLC-GFP-luc cells treated with DOX (3 μM for 4 h) ± ABCB1 inhibitor Tariquidar (Tari, 0.1 μM), analyzed by confocal microscopy. Median ± interquartile range. A representative result from two independent experiments is shown. **(b)** Quantification of ABCB1 expression detected by Western-blotting in **Fig.5c** from three independent experiments. (**c**) DOX-induced apoptosis of LLC-GFP-luc cells with addition of $ZnCl_2$. LLC-GFP-luc tumor cells were treated with 3 μM DOX combined with different concentrations of $ZnCl_2$ for 24 h in DMEM+10%FBS+5 μM TPEN. A representative result from two independent experiments is shown. **(d, e)** Quantification of ABCB1 expression detected by Western-blotting in **Fig.5g, h** from three (**5g**) or four (**5h**) independent experiments is shown. (**f**) ABCB1 expression on the cell surface of LLC-GFP-luc tumor cells treated with $ZnCl_2$ for 4 h determined by FACS. LLC-GFP-luc cells were treated with indicated conditions. TPEN, 5 μM; $ZnCl_2$, 30 μM. n = 3 for each group. **(g)** Quantification of ABCB1 expression detected by Western-blotting in **Fig.5k** from three independent experiments is shown. Unpaired *t*-test or Mann Whitney test (**e**) for two group comparison. Source data are provided as a Source Data file (**a-g**).

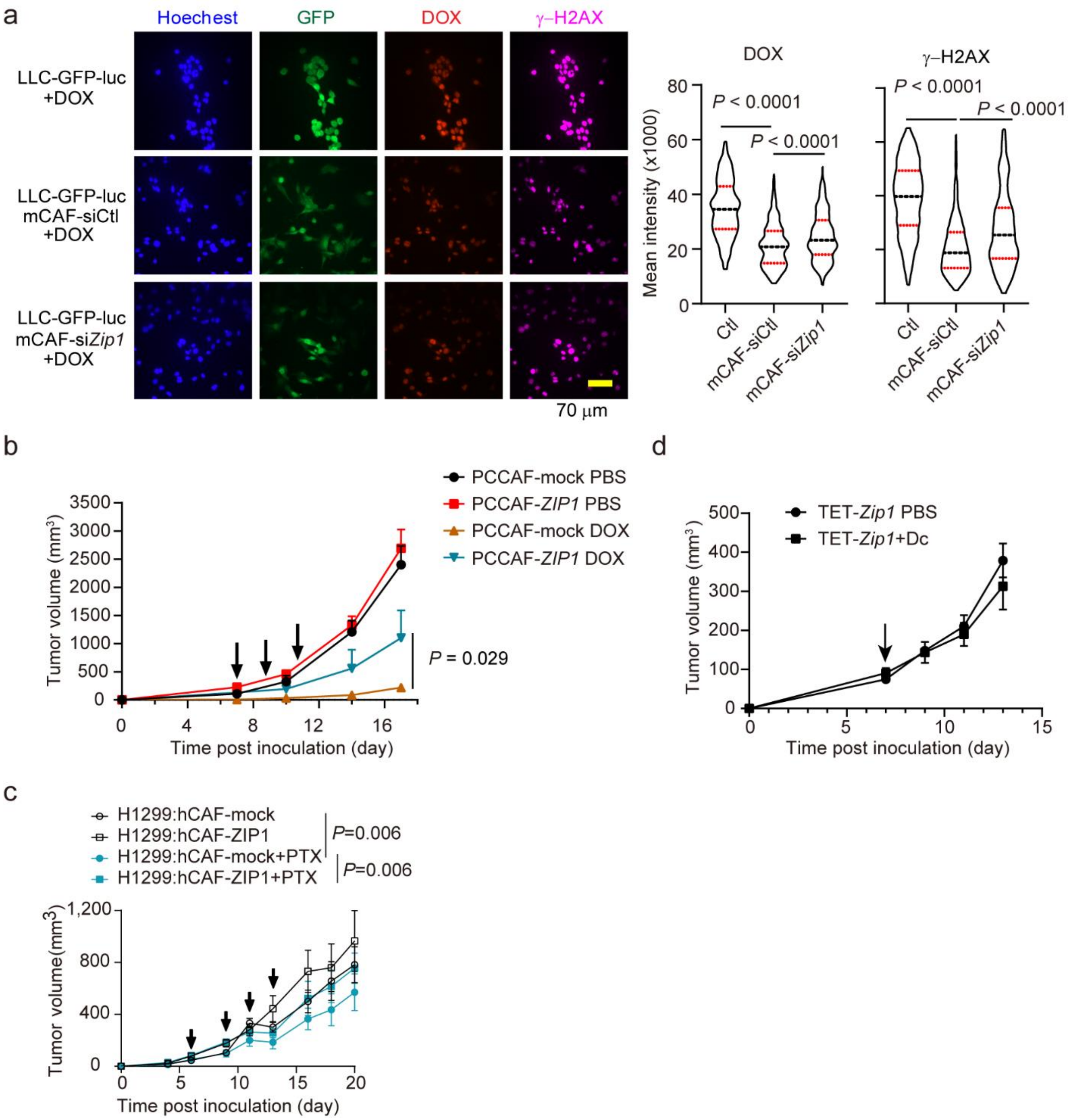
a
Hoechest
GFP
DOX
γ–H2AX
LLC-GFP-luc
+DOX
LLC-GFP-luc
mCAF-siCtl
+DOX
LLC-GFP-luc
mCAF-siZip1
+DOX
70 μm
DOX
γ–H2AX
Mean intensity (x1000)
0
20
40
60
80
P < 0.0001
P < 0.0001
P < 0.0001
P < 0.0001
Ctl
mCAF-siCtl
mCAF-siZip1
Ctl
mCAF-siCtl
mCAF-siZip1
b
PCCAF-mock PBS
PCCAF-ZIP1 PBS
PCCAF-mock DOX
PCCAF-ZIP1 DOX
Tumor volume (mm³)
0
500
1000
1500
2000
2500
3000
3500
P = 0.029
0
4
8
12
16
Time post inoculation (day)
d
TET-Zip1 PBS
TET-Zip1+Dc
Tumor volume (mm³)
0
100
200
300
400
500
0
5
10
15
Time post inoculation (day)
c
H1299:hCAF-mock
H1299:hCAF-ZIP1
H1299:hCAF-mock+PTX
H1299:hCAF-ZIP1+PTX
P=0.006
P=0.006
Tumor volume(mm³)
0
400
800
1,200
0
5
10
15
20
Time post inoculation (day)

Supplementary Figure 6. ZIP1$^+$ fibroblasts promote tumor chemoresistance. (**a**) DOX accumulation and DNA damage (indicated by γ-H2AX expression) in LLC-GFP-luc cells co-cultured with siRNA-transfected mCAF-si*Zip1* or mCAF-siCtl in DMEM+10%FBS+5 μM TPEN, analyzed by confocal microscopy. DOX (3 μM) was added to treat the cells for 4 h, and the cells were stained for GFP and γ-H2AX to identify tumor cells and DNA damage. Median ± interquartile range. Unpaired *t*-test for two group comparison. A representative result from two independent experiments is shown. (**b**) Tumor growth curve of A549 cells co-injected into mice with PCCAF-mock or PCCAF-*ZIP1* cells, ± DOX treatment. PCCAF-mock or PCCAF-*ZIP1* (9×10^5) cells were co-injected with A549 cells (3×10^5) subcutaneously into Balb/c nude mice. When the tumor volume reached ~100 mm^3, tumors were treated with DOX (5 mg/kg) every 2 days, for three treatments. n = 4 for PCCAF-mock group, n = 5 for other groups. (**c**) Tumor growth curve of H1299 cells co-injected with hCAF-mock or hCAF-*ZIP1* cells into NOD-SCID mice, ± PTX treatment. hCAF-mock or hCAF-*ZIP1* (1×10^6) cells were co-injected with H1299 cells (5×10^5) subcutaneously into NOD-SCID mice. When the tumor volume reached ~100 mm^3, tumors were treated with PTX (10 mg/kg) on indicated day for four times. n = 8 for vehicle groups, n = 7 for PTX groups. (**d**) Tumor growth curves for mice inoculated with LLC cells and mCAFs with tetracycline (Tet)-off system-controlled expression of *Zip1* (TET-*Zip1*). Arrow, doxycycline (Dc) or PBS drinking. TET-*Zip1*, co-transfected with pTet-off and pTRE2-mZip1. n = 5 for PBS group, n = 6 for Dc group. Two-way ANOVA for tumor growth curve comparison. Source data are provided as a Source Data file (**a-d**).

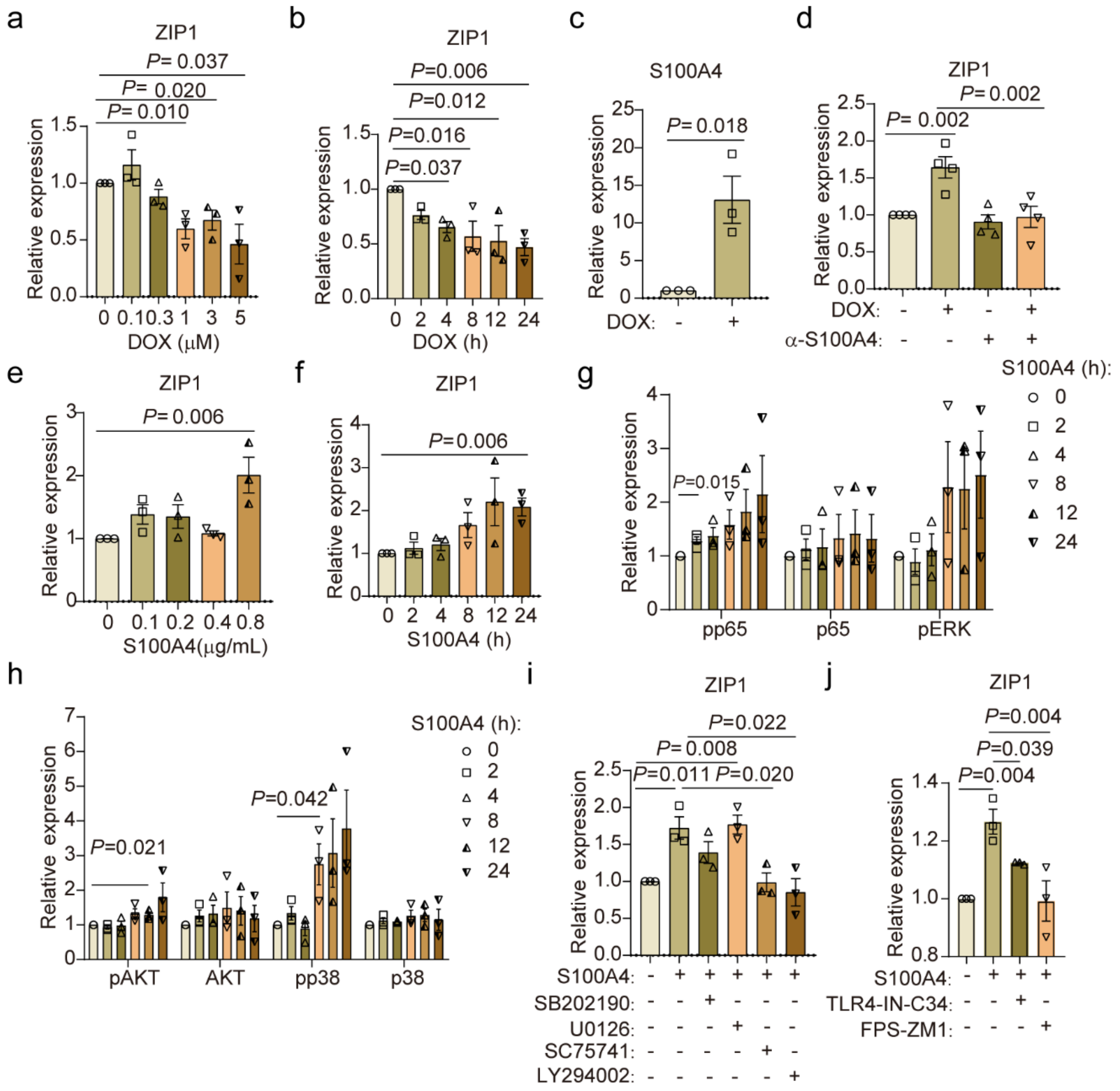

Supplementary Figure 7. S100A4 increases ZIP1 expression in fibroblasts. (a, b) Quantification of ZIP1 expression detected by Western-blotting in **Fig.7a, b** from three (**7a, b**) independent experiments is shown. **(c)** Quantification of S100A4 expression detected by Western-blotting in **Fig.7c** from three independent experiments is shown. CM without DOX treatment was used as controls. **(d)** Quantification of ZIP1 expression detected by Western-blotting in **Fig.7d** from four independent experiments is shown. **(e, f)** Quantification of ZIP1 expression detected by Western-blotting in **Fig.7e, f** from three (**7e, f**) independent experiments is shown. **(g)** Quantification of pp65, p65 and pERK expression detected by Western-blotting in **Fig.7g** from three independent experiments is shown. **(h)** Quantification of pAKT, AKT, pp38 and p38 expression detected by Western-blotting in **Fig.7h** from three independent experiments is shown. **(i, j)** Quantification of ZIP1 expression detected by Western-blotting in **Fig.7i, j** from three **(7i, j)** independent experiments is shown. Unpaired *t*-test for two group comparison. Source data are provided as a Source Data file (**a-j**).

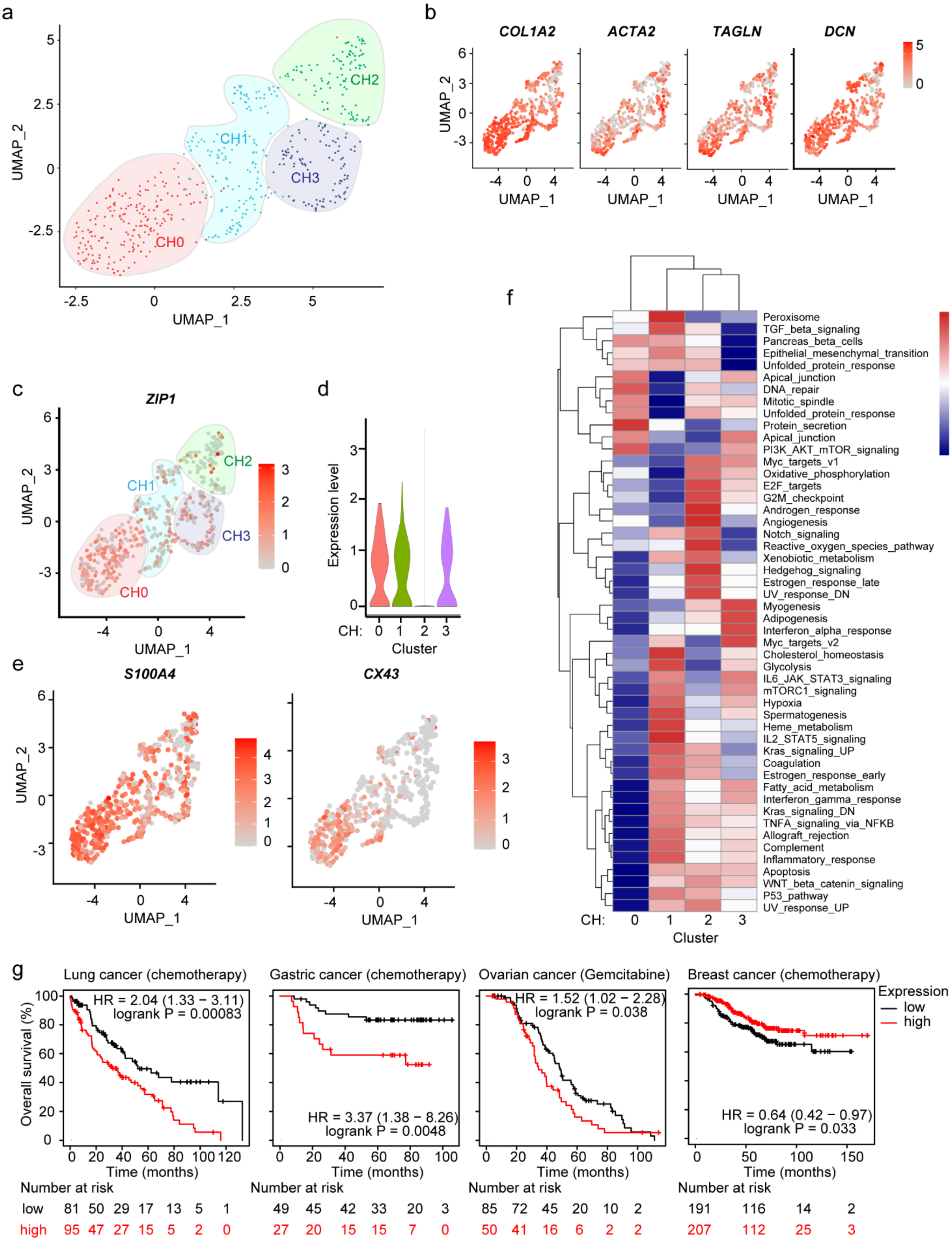
a
CH0
CH1
CH2
CH3
UMAP_1
UMAP_2
b
COL1A2
ACTA2
TAGLN
DCN
c
ZIP1
d
Expression level
CH: 0 1 2 3
Cluster
e
S100A4
CX43
f
Peroxisome
TGF_beta_signaling
Pancreas_beta_cells
Epithelial_mesenchymal_transition
Unfolded_protein_response
Apical_junction
DNA_repair
Mitotic_spindle
Unfolded_protein_response
Protein_secretion
Apical_junction
PI3K_AKT_mTOR_signaling
Myc_targets_v1
Oxidative_phosphorylation
E2F_targets
G2M_checkpoint
Androgen_response
Angiogenesis
Notch_signaling
Reactive_oxygen_species_pathway
Xenobiotic_metabolism
Hedgehog_signaling
Estrogen_response_late
UV_response_DN
Myogenesis
Adipogenesis
Interferon_alpha_response
Myc_targets_v2
Cholesterol_homeostasis
Glycolysis
IL6_JAK_STAT3_signaling
mTORC1_signaling
Hypoxia
Spermatogenesis
Heme_metabolism
IL2_STAT5_signaling
Kras_signaling_UP
Coagulation
Estrogen_response_early
Fatty_acid_metabolism
Interferon_gamma_response
Kras_signaling_DN
TNFA_signaling_via_NFKB
Allograft_rejection
Complement
Inflammatory_response
Apoptosis
WNT_beta_catenin_signaling
P53_pathway
UV_response_UP
g
Lung cancer (chemotherapy)
HR = 2.04 (1.33 − 3.11)
logrank P = 0.00083
Gastric cancer (chemotherapy)
HR = 3.37 (1.38 − 8.26)
logrank P = 0.0048
Ovarian cancer (Gemcitabine)
HR = 1.52 (1.02 − 2.28)
logrank P = 0.038
Breast cancer (chemotherapy)
HR = 0.64 (0.42 − 0.97)
logrank P = 0.033
Expression
low
high
Overall survival (%)
Time (months)
Number at risk
low 81 50 29 17 13 5 1
high 95 47 27 15 5 2 0
49 45 42 33 20 3
27 20 15 15 7 0
85 72 45 20 10 2
50 41 16 6 2 2
191 116 14 2
207 112 25 3

Supplementary Figure 8. Analysis of scRNAseq datasets of human lung adenocarcinoma identifies ZIP1^{+}S100A4^{+}CX43^{high} fibroblasts. (**a**) scRNAseq datasets of eight human primary lung adenocarcinomas (GEO: GSE123904) were analyzed and CAF cells were re-clustered. UMAP plot displaying 554 fibroblast cells classified into four subsets. (**b**) UMAP plots showing the expression of common fibroblast marker genes (*COL1A2*, *ACTA2*, *TAGLN*, *DCN*). (**c, d**) UMAP plot (**c**) and violin plot (**d**) showing the expression of *ZIP1* in fibroblast clusters. (**e**) UMAP plots showing the expression of *S100A4* and *CX43*. (**f**) Heatmap of gene-set variation analysis of hallmark signaling pathway terms in the four fibroblast subsets. (**g**) The association of the expression level of three genes (*ZIP1*, *S100A4*, *CX43*) with overall survival rate of lung, gastric, ovarian and breast cancer patients after chemotherapy. Data are derived from Kaplan-Meier plotter (kmplot.com/analysis). Log-rank test for survival curve comparison. Source data are provided as a Source Data file (**d, g**).

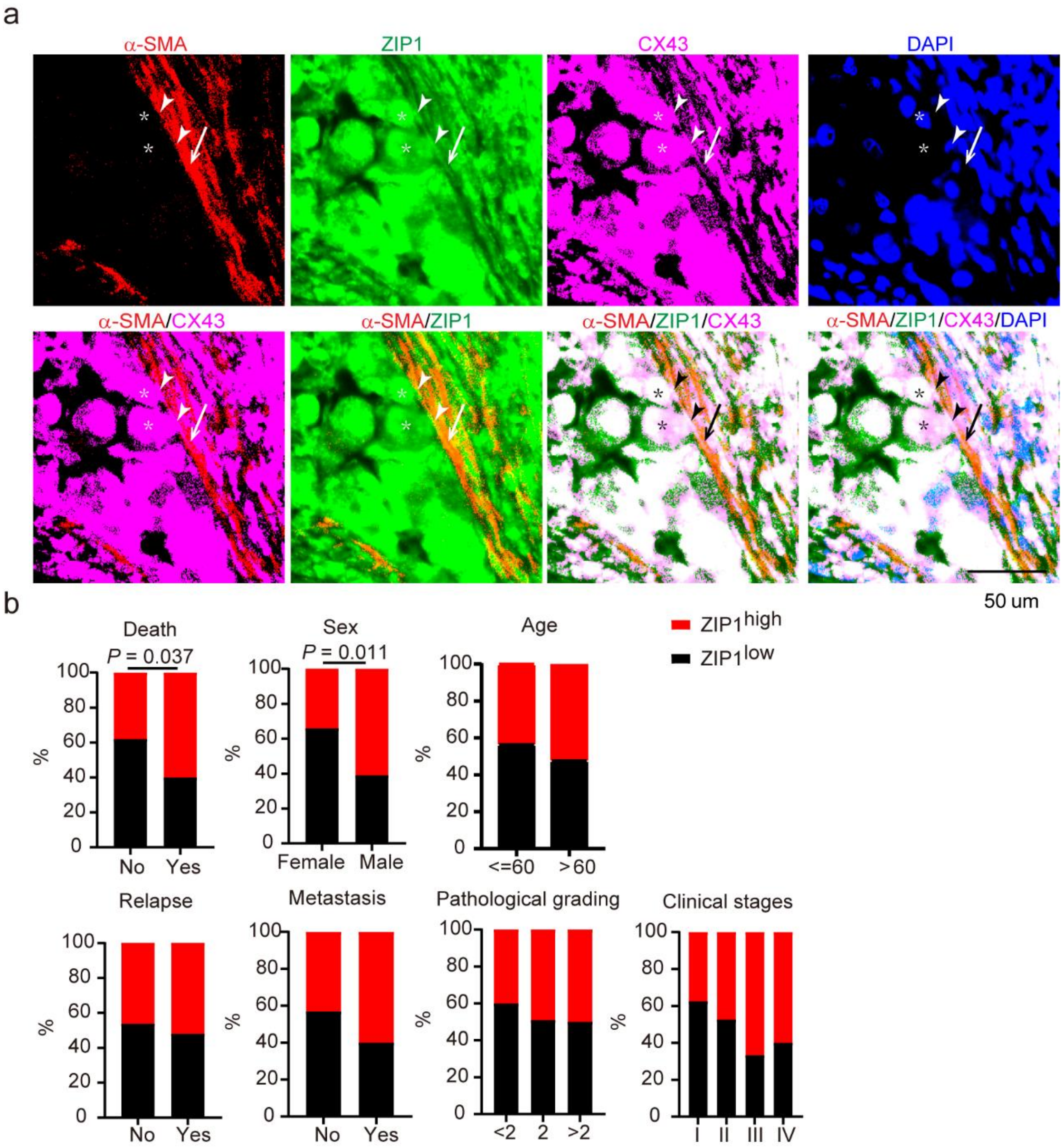

Supplementary Figure 9. ZIP1$^+$ fibroblasts interconnect cancer cells in human lung adenocarcinoma. (**a**) Representative results showing gap junctions between ZIP1$^+$ fibroblasts and cancer cells in human lung adenocarcinoma from two independent experiments are shown. Tumor sections were stained with α-SMA, ZIP1, CX43 and DAPI. Arrowhead, gap junction. Asterisk, cancer cell. Arrow, ZIP1$^+$ fibroblasts. (**b**) Relationships of ZIP1^{high} and ZIP1^{low} fibroblast status with different clinical parameters. 90 patients were analysed. The differences were analyzed by χ^2 tests. Source data are provided as a Source Data file (**b**).

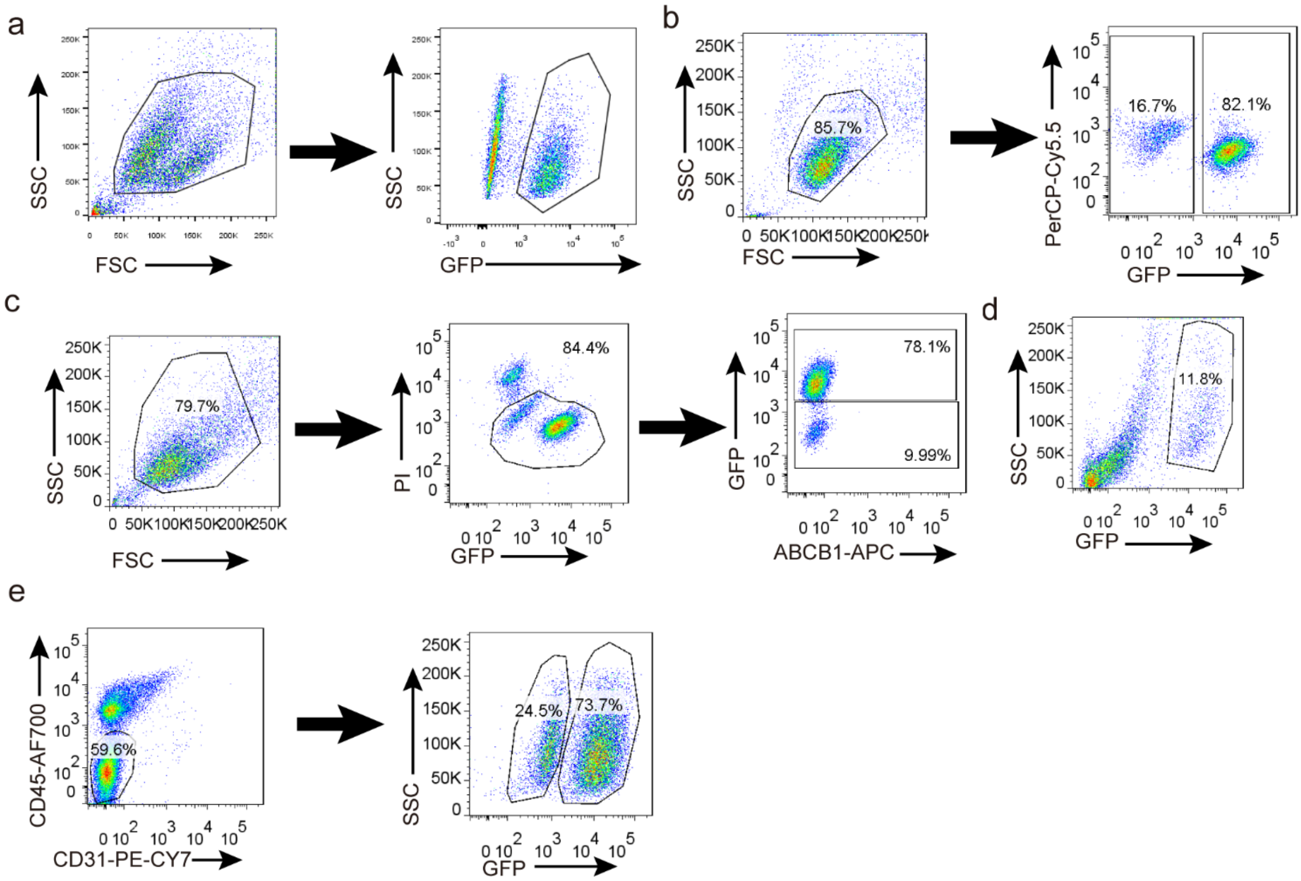

Supplementary Figure 10. FACS gating strategies. Panels (**a-e**) correspond to FACS data panels in **Figure 6a-e**, respectively.

Supplementary Table 1. Cluster characteristics. Clusters, the cell number in individual cluster, the cell number in different samples, the average number of detected genes in individual cluster, the average number of UMIs in individual cluster.

Cluster	Cells	PBS	DOX	Genes (Mean)	UMI (Mean)	Marker Genes
0	7681	1915	5766	2597	10595	*Actb,Abhd2,Notch2,Slc39a1*
1	4154	2447	1707	3713	21299	*NA*
2	3779	2244	1535	3454	16679	*Col3a1,Malat1,Dcn,Fn1,Neat1,Dpt,Prg4,Wls,Fstl1,Dkk2,Matn2,Sat1,Nupr1,Gadd45a,Jund,Nav1,Ghr,Thbs2,Il33,Angptl7,Nnmt,Dock10,Lrp1,Hk2,Osr2,Aspn,Vcan,Cebpd,Igfbp4,Sox4,Man1a*
3	3082	1819	1263	4111	25167	*Hmgb2,H2afz,Birc5,Tuba1b,Pimreg,Cenpf,Cenpa,Cdca3,Racgap1,Cdca8,Hmgb1,Ccna2,Cks2,Ccnb1,Top2a,Smc4,Stmn1,Tpx2,Cks1b,Ccnb2,Cdc20,Pclaf,Cdk1,Tk1,Pbk,Ube2c,Smc2,Selenoh,Kifc1,Nusap1,Cenpq,Kif22,Hmgn2,Mki67,Kif2c,H2afx,Mad2l1,Shcbp1,Cenpe,Tubb6,Ndc80,Aurkb,Ran,Hmgb3,Asf1b,Cenpw,Lockd,Lsm2,Kif23,Incenp,Spc24,Prc1,Anp32e,Pmf1,Tubb4b,Hmmr,Kif20a,Ube2s,Trip13,Ckap2,Nucks1,Rrm1,Gmnn,Plk4,Hmgn1,Sgo1,Knstrn,Kif20b,Usp1,Pttg1,Lmnb1,Tubb5,Cenph,Lig1,Msln,Knl1,Phgdh,Lyar,Spc25,Tcof1,Mis18bp1,Ddx39,Nrm,Plk1,Dnph1,Psat1,Reep4,Ranbp1,Bok,Srsf7,Tuba1c,Calm1*
4	394	300	94	2409	9558	*Srgn,C1qb,Fcer1g,Ctss,Tyrobp,Cd52,Ecm1,Arg1,Lyz2,C1qc,C1qa,Ms4a8a,Pf4,Saa3,Cxcl2,Ltc4s,Ccl6,Wfdc17,Laptm5,Mrc1,Csf2rb,Alox5ap,Fxyd5,Sh3bgrl3,Wfdc21,Cotl1,Apoe,Fcna,F13a1,Coro1a,Ucp2,C5ar1,Ms4a7,Ccl9,Cd33,Mafb,P2ry6,S100a8,Rac2,Fcgr2b,Fcgr3,Clec4d,Psmb8,Lgmn,Bcl2a1b,Spi1,Ms4a6c,Smpdl3a,AA467197,Slfn2,Cd68,Cxcl3,Cxcl16,Itgam,Lcp1,Cd300lf,Cfp,Ncf4,Rgs1,Tgfbi,Ptpn18,Pmepa1,Itgb2,Ctsh*
5	390	127	263	2863	10952	*Tmem252,Plvap,Ctla2a,Igfbp3,Gpihbp1,Lrg1,Aqp1,Cldn5,8430408G22Rik,Flt1,Egfl7,Cdh5,H2-K1,Ly6a,Ecscr,Pecam1,Pdlim1,Ly6c1,Trp53i11,Gda,Adgrf5,Scarb1,Cd93,Mmrn2,Csrp2,Rtl8a,Cd34,Nfkbia,Upp1,Cd300lg,Rbp1,S1pr1,Ptprb,Jam2,Tnfrsf9,Tspan13,Ldb2,Cd36,Prss23,Rasip1,Tspan15,Mfng,Adgrg1,Rnd1,Tgm2,Car4,Prex2,Tm4sf1,Grrp1,Vwf,Cyyr1*
6	73	69	4	2951	10773	*Meg3,Rgs5,Gja4,Cyp4b1,Sparcl1,Ednrb,Sparc,Gm12840,Igfbp7,Nr4a1,Hspb1,Timp3,Rasl11a,Gm13889,Steap4,Rgs2,Ndufa4l2,Col5a2,Cryab,Rarres2,Cd302,Gimap6,Adamts4,Lama2,Rasd1,Hrct1,Esam,Loxl1,Cdc42ep3,Fabp4,Col4a1,Inhbb,Spon1,Gpx7,Pdgfrb,Il6,Ramp1,Thbs1,Col4a2,Emid1,Cd53,Jaml,Slc12a2,Gadd45b,Pid1,Acer2,Sult1a1,Olfml2b,Pde4b,Emcn,F11r,Angpt2,Apod,Clca3a1,Hspa1a,Adamts9,Pdgfrl,Tnfaip3,Ebf1,Fam43a,Slfn5,Cd200,Uchl1,Gpx3,Tnfaip6,Postn,Angptl4,Cd248,Plpp3,Ndn,Slc11a1,Pmp22,Serping1,Des,Smagp,Serpine2,Selenbp1,Btg2,Mustn1,Iigp1,Itga1,Ass1,Tinagl1,Bgn,Col6a3,Adamts1,Pik3r1,App,Cystm1,Gngt2,Kcnj8,Lysmd2,Atf3,Selenop,Crip2,Isg15,Anxa6,Tnfrsf23,Cdkn1a,Cygb,Zfp36l1,Gm42418,Crip1,Tmsb4x,Nr4a2,Ece1,Pla1a,Gpm6b,Id3,Bcam,Pycard,P4ha2,Egr1,Junb,Gas1,Arhgdib,Gng11,Hacd2,Sept4,Gadd45g,Id1,Hes1,Cfh,Enah,Metrnl,Tmem176b,Hilpda,Ppp1r14a,Fosb,Plin2,Tsc22d1,Plat,Cp,Apold1,Jun,H2-D1,Fos,Pdgfa,Marcks,Dnajb1,Tacc3,Dennd5b,mt-Nd1,Dusp1,Rasgrp2,Trib1,Spry2,Cpe,Acta2,Mcam,Lgals9,Tmem176a,Ifitm1,Loxl2,Rhob,Ddit4,Dusp2,mt-Cytb,Mndal,Rgs16,Myl9,mt-Co2,Phlda1,Exosc8*
7	64	43	21	2764	10659	*Col1a1,Rnase4,Ctsk,Mmp2,Col1a2,Nbl1,Htra3,Serpina3n,Mfap5,Hsd11b1,Fbln2,Timp1,Cilp,Wisp2,Tnfrsf26,Sema3c,Il11ra1,Plac8,Crispld2,S100a16,Fbn1,Lrrn4cl,Tmem119,Rora,Lsp1,Pdpn,Fam46a,Gas7,a,Col6a2,Olfml3,Klf4*

Supplementary Table 2. The top ranked 100 genes for each gene expression programs (GEPs) based on the gene_spectra_score of cNMF.

GEP	Top ranked 100 genes
GEP1	*Mt2, Fth1, Notch2, Mt1, Pam, Tsc22d3, Cirbp, Spry2, Sdc4, Actb, Abhd2, Anxa1, Ftl1, Cp, Eef2, Nfia, Slc39a1, Fkbp5, Hsp90ab1, Aplp2, Prune2, Gpnmb, Ubb, Cbx3, Rpl10, Dusp1, Txnip, Rps26, Rps4x, Sgk1, Cxcl1, Gnb1, Tpt1, Rplp0, Preb, Prdx6, Angptl7, Wtap, Ezr, Klf9, Insig1, Eef1a1, Fos, Lgals3, Kdm6b, Rps27a, Rpl12, Rps24, Hes1, Ddx5, Srrm2, Rps12, Pan3, Rps8, Rpl21, Sqstm1, Rpl29, Rps9, Rpl18a, Lrrc8a, Pfdn5, Rps20, Rpl32, Alkbh5, S100a4, Psap, Pabpc1, Kdelr2, Rpl4, Pdcd4, Rhoa, Eif4ebp1, Rps27, Fhl3, Cdkn1b, Rps25, Rps3a1, Rps29, Rps10, Sept10, Rplp1, Junb, Gsk3b, Anxa2, Rpl17, Cox7a2l, S100a11, Rpl36a, Rpl30, Klf13, Klf2, Fau, Sik1, Rpl36, Rps14, Eif4a1, Rps23, Socs3, Itm2b, Rps7*
GEP2	*Gchfr, Mif, AY036118, Ldha, Hspe1, Bst2, Ung, Tpm1, Dctpp1, Fam162a, Bsg, Uqcc2, Gm42418, Aldoa, Gm13889, Rrm2b, Fdps, Pfkl, Sqle, Ppid, Gm29666, Cox8a, Cisd3, Ppil1, Prss22, Ndufb2, Slc2a1, AC160336.1, Wdcp, Cycs, Hbegf, Fxyd5, Idi1, Hells, Pgk1, Uchl3, Areg, Serf2, Pthlh, Ifitm3, Aldh1a3, Uqcrq, Higd1a, Acat2, Sdf2l1, Edf1, Cnn2, Rpl13a, Cox17, Cyp51, Ydjc, Gamt, B2m, Fgfbp3, Nop10, Ndufv3, Nptx1, Nme1, Ly6c1, Tma7, Ndufa13, Ankrd37,Txnl4a, Fasn, Olfr889, Egfl7, Mrps6, Gstt1, Sigmar1, Tpi1, Nudt5, Scd2, Mydgf, Pgam1, Ccne2, Uqcr11, Pmvk, Naa38, Cisd1, Ero1l, Cyb5b, Coa3, Tfrc, Pbld1, Sms, Tmem80, Tomm5, Gale, Snrpe, Mrpl14, Plec, Odc1, Gch1, Mgst3, 2010107E04Rik, Ccne1, Mcrip2, 2900009J06Rik, Dbi, Fam96b*
GEP3	*Dcn, Lum, Dpt, Col3a1, Fn1, Fbn1, Bgn, Gng11, Col6a3, Mfap4, Mmp3, Aspn, Dkk2, Sparc, Fap, Vcan, Sat1, Ghr, Nupr1, Mmp12, Prg4, Ccl7, Cfh, Thbs2, Enpp2, Anxa6, Fstl1, Adamts1, Nnmt, Ebf1, Zbp1, S100a16, Nfkbiz, Sdc2, Maged2, Ier2, Phlda1, Hk2, Gpr153, Il33, Igfbp4, Ccl8, Ccl11, Egr1, Tnc, Cpq, Neat1, Vegfd, Wls, Plod2, Man1a, Copz2, Prrx1, Rgma, Lsp1, Cdon, Gadd45a, Lrp1, Col4a5, Ptn, Zbtb20, Col1a1, Nav1, Nr2f1, Itm2b, Nrp1, P3h4, Inhba, Lrrc17, AC149090.1, Lpar1, Wisp1, Ptgfrn, Ass1, Fkbp10, Gpm6b, Antxr1, Trp53i11, Hist1h1c, Mmp14, Osr2, Matn2, Tmed3, Pros1, Snhg18, Ifi207, Jund, Scarb2, Cilp, Medag, Kdelr3, Fcgrt, Mxra8, Malat1, Hexa, B3gnt9, Selenom, Ifi27, Klf6, Rgmb*
GEP4	*Birc5, Ube2c, Hmgb2, Ccnb1, Ccna2, Cdca8, Pimreg, Cenpf, Cdc20, Cdca3, Cenpa, Tpx2, Prc1, Top2a, Nusap1, Cenpe, Plk1, Mki67, Aurka, Hmmr, Racgap1, Cdk1, Kif23, Cks2, Aurkb, Smc4, Smc2, Kif22, Ccnb2, Tacc3, Pclaf, Stmn1, Sgo1, Spc25, Kifc1, H2afx, Knl1, Kif20b, Kif20a, Mis18bp1, Kif2c, Ckap2l, Ndc80, H2afz, Pbk, Tuba1b, Knstrn, Incenp, Sapcd2, Spc24, Asf1b, Mad2l1, Cenpq, Sgo2a, Lockd, Shcbp1, Cks1b, Cdkn3, Tubb4b, Ska1, Tk1, Cep55, Cenpw, Aspm, Nek2, Ckap2, Dlgap5, Anln, Hmgn2, Cit, Bub1, Ncapg, Mxd3, Depdc1a, Kif11, Cdca2, Cdc25c, Pttg1, Hmgb3, Troap, Hmgb1, Bub1b, Nuf2, Ect2, Tubb6, Pmf1, Selenoh, Cdca5, Ube2s, Kpna2, Ccnf, Trip13, Gas2l3, Dbf4, Lsm2, Melk, Ncapd2, Lmnb1, Kif4 Kif15*

Supplementary Table 3. Selected genes upregulated in the transition from Cluster 0 to Cluster 2 and within Cluster 0.

Cluster transition	Upregulated genes
Cluster 0 to Cluster 2	*Wls, Nnmt, Plod2, Vcan, Col3a1, Ebf1, Fstl1, Sdc2, Dcn*
Cluster 0 to Cluster 0	*Spry2, Cxcl1, Alkbh5, Mt2, Mt1, Nfia*

Supplementary Table 4. Patient information of 30 lung adenocarcinoma cases.

Patient ID	Histolgy	Pathological stage	Biopsy Site
E05A3080	Adenocarcinoma	Ⅰ - Ⅱ	Lung
E05A3302	Adenocarcinoma	Ⅰ - Ⅱ	Lung
E05A3459	Adenocarcinoma	Ⅰ - Ⅱ	Lung
E05A3072	Adenocarcinoma	Ⅱ	Lung
E05A3081	Adenocarcinoma	Ⅱ	Lung
E05A3086	Adenocarcinoma	Ⅱ	Lung
E05A3095	Adenocarcinoma	Ⅱ	Lung
E05A3305	Adenocarcinoma	Ⅱ	Lung
E05A3315	Adenocarcinoma	Ⅱ	Lung
E05A3362	Adenocarcinoma	Ⅱ	Lung
E05A3374	Adenocarcinoma	Ⅱ	Lung
E05A3416	Adenocarcinoma	Ⅱ	Lung
E05A3478	Adenocarcinoma	Ⅱ	Lung
E05A3723	Adenocarcinoma	Ⅱ	Lung
E05A3093	Adenocarcinoma	Ⅱ-Ⅲ	Lung
E05A3111	Adenocarcinoma	Ⅱ-Ⅲ	Lung
E05A3234	Adenocarcinoma	Ⅱ-Ⅲ	Lung
E05A3238	Adenocarcinoma	Ⅱ-Ⅲ	Lung
E05A3247	Adenocarcinoma	Ⅱ-Ⅲ	Lung
E05A3264	Adenocarcinoma	Ⅱ-Ⅲ	Lung
E05A3327	Adenocarcinoma	Ⅱ-Ⅲ	Lung
E05A3363	Adenocarcinoma	Ⅱ-Ⅲ	Lung
E05A3369	Adenocarcinoma	Ⅱ-Ⅲ	Lung
E05A3370	Adenocarcinoma	Ⅱ-Ⅲ	Lung
E05A3424	Adenocarcinoma	Ⅱ-Ⅲ	Lung
E05A3804	Adenocarcinoma	Ⅱ-Ⅲ	Lung
E05A4547	Adenocarcinoma	Ⅱ-Ⅲ	Lung
E05A3101	Adenocarcinoma	Ⅲ	Lung
E05A3300	Adenocarcinoma	Ⅲ	Lung
E05A3301	Adenocarcinoma	Ⅲ	Lung
