# Supplementary material for: ZIP1+ fibroblasts protect lung cancer against chemotherapy via connexin-43 mediated intercellular Zn2+ transfer
Source: Nat Commun. 2022 Oct 7;13:5919. doi: 10.1038/s41467-022-33521-4 (PMC9547061; doi:10.1038/s41467-022-33521-4)
Supplement: Supplementary file 3 — Description of Additional Supplementary Files [file 41467_2022_33521_MOESM3_ESM.pdf]

Supplementary Data 1: Enrichment analysis of whole clusters-specific markers based on current pathway databases (KEGG and GO).

Supplementary Data 2: GO enrichment analysis of top 100 genes in GEP1-4.

Supplementary Data 3: Marker genes of CAF subclusters from human lung adenocarcinoma (GSE123904).

Supplementary Data 4: Enrichment analysis of whole clusters-specific markers of human lung CAF (GSE123904) based on current pathway databases (GO).

Supplementary Data 5: Patient information of 90 lung adenocarcinoma cases.
